# Pharmacogenetic Profiling in High-Risk Soft Tissue Sarcomas Treated with Neoadjuvant Chemotherapy

**DOI:** 10.3390/jpm12040618

**Published:** 2022-04-11

**Authors:** Anna C. Virgili Manrique, Juliana Salazar, María Jesús Arranz, Silvia Bagué, Ruth Orellana, Antonio López-Pousa, Paula Cerdà, Isidre Gracia, Katarina Majercakova, Ana Peiró, Laura Trullols, Manuel Fernández, Sandra Valverde, María Jesús Quintana, Olga Bell, Alícia Artigas-Baleri, Ana Sebio

**Affiliations:** 1 Department of Medical Oncology, Hospital de la Santa Creu i Sant Pau, 08041 Barcelona, Spain; avirgili@santpau.cat (A.C.V.M.); alopezp@santpau.cat (A.L.-P.); pcerda@santpau.cat (P.C.); 2 Department of Medicine, Universitat Autònoma de Barcelona, 08035 Barcelona, Spain; 3 Medical Translational Oncology Laboratory, IIB-Sant Pau, Hospital de la Santa Creu i Sant Pau, 08041 Barcelona, Spain; obell@santpau.cat; 4 Fundació Docència i Recerca Mútua Terrassa, 08221 Terrassa, Spain; mjarranzc@gmail.com; 5 Department of Pathology, Hospital de la Santa Creu i Sant Pau, 08041 Barcelona, Spain; sbaguer@santpau.cat (S.B.); rorellana@santpau.cat (R.O.); 6 Orthopaedics and Trauma Surgery, Hospital de la Santa Creu i Sant Pau, 08041 Barcelona, Spain; igracia@santpau.cat (I.G.); apeiro@santpau.cat (A.P.); ltrullols@santpau.cat (L.T.); 7 Radiation Oncology, Hospital de la Santa Creu i Sant Pau, 08041 Barcelona, Spain; kmajercakova@santpau.cat; 8 Plastic and Reconstructive Surgery, Hospital de la Santa Creu i Sant Pau, 08041 Barcelona, Spain; mfernandezga@santpau.cat; 9 Radiology Department, Hospital de la Santa Creu i Sant Pau, 08041 Barcelona, Spain; svalverde@santpau.cat; 10 Epidemiology Department, Hospital de la Santa Creu i Sant Pau, 08041 Barcelona, Spain; mjquintana@santpau.cat; 11 Genetics Department, IIB-Sant Pau, Hospital de la Santa Creu i Sant Pau, 08041 Barcelona, Spain; aartigas@santpau.cat

**Keywords:** soft tissue sarcoma, neoadjuvant, pharmacogenetics, anthracyclines, ifosfamide, *ALDH1A1*, *ABCC2*, *ABCB1*

## Abstract

Neoadjuvant chemotherapy based on anthracyclines and ifosfamide for high-risk soft tissue sarcomas (STS) of the extremities and trunk is a controversial treatment option. There are substantial interindividual differences in clinical outcomes in patients treated with neoadjuvant chemotherapy. The aim of this study was to evaluate, as biomarkers, polymorphisms in genes encoding drug-metabolizing enzymes, drug transporters, or drug targets and their association with toxicity and survival in STS patients treated with neoadjuvant chemotherapy. We analysed variants in genes involved in anthracycline metabolism (*ABCB1*, *ABCC2*, *NQO1*, *CBR3*, and *SLC22A16*) and in ifosfamide catabolism (*ALDH1A1*) in 79 treated patients. Two genes showed significant association after adjusted multivariate analysis: *ABCC2* and *ALDH1A1*. In patients treated with anthracyclines, *ABCC2* rs3740066 was associated with risk of febrile neutropenia (*p* = 0.031), and with decreased overall survival (OS) (*p* = 0.024). *ABCC2* rs2273697 was associated with recurrence-free survival (RFS) (*p* = 0.024). In patients treated with ifosfamide, *ALDH1A1* rs3764435 was associated with RFS (*p* = 0.046). Our pharmacogenetic study shows for the first time that variants in genes regulating the metabolism of neoadjuvant chemotherapy may be helpful to predict toxicity and survival benefit in high-risk STS treated with neoadjuvant chemotherapy. Further validation studies are needed to establish their clinical utility.

## 1. Introduction

Soft tissue sarcomas (STS) are a group of rare diseases that include more than 80 different subtypes [1]. Wide local excision is the gold-standard treatment. Nonetheless, approximately 50–60% of patients diagnosed with the high-risk localised disease will develop distant metastases despite appropriate surgery, and long-term survival is poor. Perioperative chemotherapy and/or radiotherapy may improve the long-term prognosis and is an option, although controversial, for patients with STS of the extremities and trunk who are considered to be at a high risk of relapse [2]. Perioperative chemotherapy consists of a combination of anthracyclines and ifosfamide. Administration in the pre-operative setting has several advantages over adjuvant treatments, such as the possibility of tumour downstaging, allowing limb-sparing surgery, early treatment of micrometastatic disease, and evaluation of tumour chemosensitivity [3]. Despite the known benefits of chemotherapy, there are huge interindividual differences in terms of toxicity and outcome. These interindividual differences are independent of patient characteristics and histology and might compromise adherence to treatment and, potentially, treatment benefit and survival. Common clinical factors used for decision making when considering neoadjuvant chemotherapy in high-risk patients include histology, age and performance status, but new biomarkers are needed to personalize treatments and reduce side effects that could jeopardize the patient’s prognosis.

The antitumour activity and toxicity of doxorubicin and its 4′-epi-isomer epirubicin may be conditioned by alterations in their transporters or in the enzymes related to the generation of free-radicals that provoke DNA and cell membrane damage [4,5]. These transporters include the ATP-binding cassette (ABC) proteins ABCB1 (P-gp, MDR1) and ABCC2 (MRP2), which are efflux transporters involving doxorubicin disposition [4,6,7], and the solute carrier SLC22A16, an organic cation influx transporter that mediates doxorubicin uptake in cancer cells [8]. Enzymes involved in doxorubicin metabolism include NAD(P)H quinone oxidoreductase I (NQO1), which is implicated in processes that protect against oxidative stress and carcinogenesis, such as stabilization of the p53 tumour suppressor [9], and the carbonyl reductases (CRBs) CBR3 and CRB1, which catalyse the reduction of doxorubicin to doxorubicin in vivo [10].

Ifosfamide is a DNA alkylating agent that is transformed into several metabolites, some being therapeutically active and others toxic. In this process, aldehyde dehydrogenase 1A1 (ALDH1A1) mediates the detoxification of aldoifosfamide to carboxyifosfamide, and modifications in this enzyme activity are known to be related to toxicity and tumour resistance [11].

Polymorphisms of genes coding for these proteins may influence the pharmacokinetic and pharmacodynamic variability of anthracycline or ifosfamide therapies, and, therefore, contribute to toxicity and treatment resistance and eventually compromise survival [12]. Several studies, mostly performed in breast cancer, have tried to correlate single nucleotide polymorphisms (SNPs) in these genes with toxicity or outcome, with inconclusive results [9,13,14,15,16,17]. Moreover, information about pharmacogenetics in sarcomas is scarce, and to our knowledge, no studies have been performed to date in the context of high-risk localised STS. The aim of this study was to analyse the relationship between germline polymorphisms in genes involved in the metabolism of anthracyclines or ifosfamide and toxicity, pathological response and survival in high-risk localised STS treated with neoadjuvant chemotherapy.

## 2. Materials and Methods

### 2.1. Study Population

We included 95 patients diagnosed with extremity or trunk STS treated with neoadjuvant chemotherapy or chemoradiotherapy at Hospital de la Santa Creu i Sant Pau from January 2006 to March 2021. Patients received different regimens of chemotherapy according to local practice or clinical trials. The majority of patients received epirubicin 60 mg/m^2^ per day (days 1, 2) plus ifosfamide 3 g/m^2^ per day (days 1, 2, 3), repeated every 21 days for 3 cycles (64.2% of patients), or high dose ifosfamide 12 g/m^2^ (25.2% of patients) (Table 1). The use of neoadjuvant or adjuvant radiotherapy was discussed individually in the multidisciplinary tumour board.

Four patients were diagnosed with stage IV disease but were treated with chemotherapy with neoadjuvant intent to pursue surgery. These patients were included in the toxicity and response analyses, but not in the survival calculations.

Of the 95 patients treated with neoadjuvant chemotherapy, for 40 patients DNA was extracted from blood samples, and for 39 patients DNA was extracted from formalin-fixed paraffin-embedded (FFPE) tumour tissue. DNA was not available for 16 patients and, therefore, they were not included in the genetic analyses.

Regarding toxicity, only grade 3 and 4 events were recorded. Toxicities were graded using CTCAE v.4.03 [18]. The major toxicities included were anaemia, thrombocytopenia, neutropenia, febrile neutropenia, transaminitis and haemorrhagic cystitis. For evaluating the pathological response, we dichotomized the variable to higher or lower than 90% response, considering necrosis and other therapy-related changes according to an adaptation of the EORTC-STBSG recommendations [19].

The study was conducted according to the guidelines of the Declaration of Helsinki, and approved by the Institutional Ethics Committee of Institut de Recerca Biomèdica Sant Pau (IIB-Sant Pau) (IIBSP-SAR-2016-102). All patients gave a signed, informed consent.

### 2.2. Genotyping

We analysed 10 SNPs in 5 genes involved in anthracycline metabolism (*ABCB1*, *ABCC2*, *NQO1*, *CBR3*, and S*LC22A16*) and 2 SNPs in the *ALDH1A1* gene involved in ifosfamide catabolism. The selected SNPs were variants with functional evidence or previously reported clinical associations with chemotherapy regimens containing anthracyclines and/or ifosfamide [4,6,11]. All of the SNPs had a minor allele frequency (MAF) over 0.15 and an r2 threshold of 0.8 in the European population [20]. Table 2 provides detailed information on the selected SNPs, and summarizes the studies with significant findings on the functionality of the SNPs or on pharmacogenetic associations.

Genomic DNA was obtained by automatic extraction from peripheral whole-blood samples (Autopure, Qiagen, Hilden, Germany) or using the GeneRead DNA FFPE Kit (Qiagen, Hilden, Germany) from FFPE tumour blocks. DNA was quantified and its quality was checked using the NanoDrop 2000 spectrophotometer (Thermo Fisher Scientific, Wilmington, DE, USA). The SNPs were analysed by real-time PCR using TaqMan^®^ SNP genotyping assays on a 7900 HT Real-Time PCR System (Applied Biosystems, Foster City, CA, USA). All the procedures were performed as specified in the manufacturers’ instructions.

We performed a genotype quality control and observed that the *CBR3* rs8133052 variant had a high missing genotype rate (>90%) and the *SLC22A16* rs12210538 showed a significant deviation from Hardy-Weinberg equilibrium, and therefore we removed both of them from the association studies. The allele frequencies of the rest of the SNPs were similar to those reported in the 1000 Genomes project for the European population. All DNA samples had a call rate higher than 90% and therefore were included in the study.

### 2.3. Statistics

Recurrence-free survival (RFS) was defined as the time from the start of neoadjuvant chemotherapy until the date of local or distant recurrence, whichever occurred first. Overall survival (OS) was calculated from the date of diagnosis (biopsy) to death from any cause or last clinical follow-up. The associations between SNPs and toxicities or responses were evaluated with cross-tables using Chi-square or Fisher’s exact test according to the variable characteristics. For the RFS and OS analyses, we used Kaplan-Meier curves and a log-rank test. Cox regression was applied for the multivariate analyses, including the statistically significant clinicopathological variables as covariables. Our sample size had over 80% statistical power to detect the effect of genetic variants with an f = 0.25 (two-sided test with α = 0.05) (G*power version 3.1.9.2, Düseldorf, Germany) [35].

All the SNPs were tested for Hardy-Weinberg equilibrium using a Chi-square test. For the tri-allelic variant *ABCB1* rs2032582, we considered the patients with GG genotype to be wild-type, patients with GT or GA genotypes to be heterozygous, and patients with TT, TA or AA genotypes to be homozygous for the low-frequency alleles, in order to enable cross-table analyses, as we did in a previous study [36]. We also removed patients with GA, TA and AA genotypes (*n* = 5) for haplotype analysis with PLINK. We considered co-dominant, dominant, and recessive models of inheritance whenever appropriate. Haplotype analyses for *ABCB1*, *ABCC2* and *ALDH1A1* were performed to explore the influence of specific allelic combinations on toxicity, pathological response and survival. Statistical significance was set at less than 0.05. Statistical analyses were performed using SPSS (version 25, IBM), and haplotype analyses using PLINK (v1.07, Shaun Purcell, http://pngu.mgh.harvard.edu/purcell/plink/ last accessed on 14 March 2022) [37].

## 3. Results

Patient and tumour characteristics are summarized in Table 1. The median OS in our series, excluding stage IV patients, was 79.7 (range 24.2–135.2) months, and the median RFS was 31.7 (range 19.1–44.3) months. Sex, age and administration of radiotherapy (RT) were found to be significantly associated with OS (*p* = 0.042, *p* = 0.022 and *p* = 0.016 respectively). These variables were included in the multivariate analyses for survival. For toxicity and response analyses, sex, age and administration of neoadjuvant radiotherapy were included in the multivariate analysis.

### 3.1. Genetic Variants and Toxicity

Fifty-four patients treated with anthracyclines and 71 treated with ifosfamide were available for toxicity analyses (Table 3).

For patients treated with anthracyclines, the *ABCC2* rs3740066 variant was significantly associated with the risk of febrile neutropenia, as 77.8% of patients (7/9) with TT genotype developed febrile neutropenia, compared to 33.3% (8/24) of heterozygous patients, and 27.8% (5/18) of patients homozygous for the most frequent C allele (*p* = 0.040). This statistical significance was maintained in the multivariate analysis (*p* = 0.031). For the *ABCC2* rs2273697, 48.6% (17/35) of GG homozygous patients developed febrile neutropenia compared to 26.7% (4/15) of heterozygous patients and no patients (0/4) with AA genotype (*p* = 0.103). In the multivariate analysis, a trend was observed toward the association between this SNP and the risk of febrile neutropenia (*p* = 0.077).

Univariate haplotype analysis including *ABCC2* variants (rs3740066|rs2273697) showed that the TG haplotype was significantly associated with febrile neutropenia (*p* = 0.040), and this association was retained in the multivariate analysis (*p* = 0.040). The same was found for the CA haplotype (univariate: *p* = 0.006 and multivariate: *p* = 0.035) (Supplementary Table S1).

When we studied *ABCB1* variants individually we obtained no associations with toxicity, although when we conducted haplotype analysis (rs1128503|rs2032582|rs1045642) the TGT haplotype was significantly associated with grade 3-4 anaemia in the multivariate analysis (*p* = 0.02) (Supplementary Table S1).

### 3.2. Genetic Variants and Survival

Fifty of the patients receiving neoadjuvant anthracycline-based chemotherapy were eligible for survival analysis (Table 4). Two polymorphisms in *ABCC2* were found to be associated with survival: rs3740066 and rs2273697.

**Figure 1:**
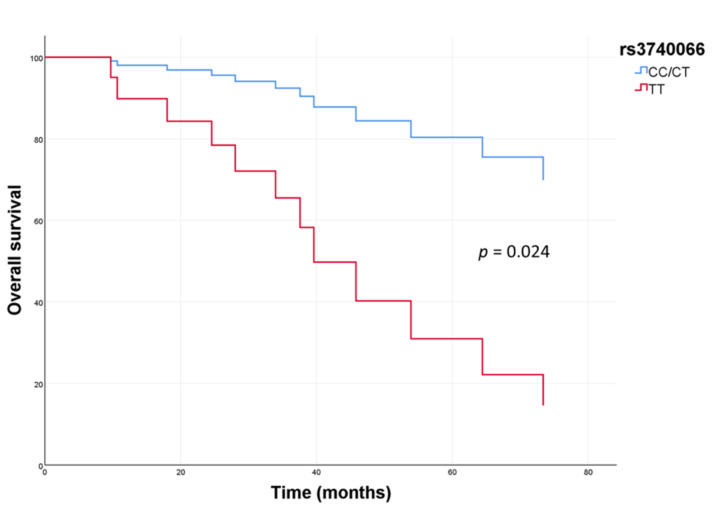
Overall survival according to *ABCC2* rs3740066 variant in patients treated with anthracyclines (multivariate analysis).

For rs3740066, the 5-year OS was 25% for patients with a TT genotype, compared to 78% for patients with CT or CC genotypes (HR: 4.4, 95% CI: 1.21–16.31; *p* = 0.014 in a recessive model). This significance was maintained in the multivariate analysis (HR: 5.4, 95% CI: 1.2–22.9; *p* = 0.024) (Figure 1). For the rs2273697 SNP, 45% of patients with GG or GA genotypes were free from recurrence at 5 years compared to 33% of patients with AA genotype (*p* = 0.042 in a recessive model), and this association was maintained in the multivariate analysis (HR: 4.6, 95% CI: 1.2–17.5; *p* = 0.024). We observed a trend toward a worse 5-year OS associated with this polymorphism (68% for GG or GA vs. 33% for AA; *p* = 0.095).

Haplotype analyses for *ABCB1* variants (rs1128503|rs2032582|rs1045642) showed that CGT (univariate: *p* = 0.042 and multivariate: *p* = 0.047) and TGT (multivariate: *p* < 0.001) haplotypes were significantly associated with OS. The haplotype TGT was also associated with RFS (univariate: *p* < 0.001 and multivariate: *p* = 0.001) (Supplementary Table S2).

Sixty-eight out of 71 patients receiving ifosfamide as part of neoadjuvant treatment were included in the survival analysis (Table 4). We observed a significant association between *ALDH1A1* rs3764435 and 5-year OS. The 5-year OS was 38% in patients with AA genotype compared to 71% in patients carrying the C allele (HR: 2.3, 95% CI: 1.02-5.17; *p* = 0.038 in a dominant model), although this significance was not maintained in the multivariate analysis (*p* = 0.095). This significant association was also observed with RFS, as patients with an AA genotype had a 5-year RFS of 25% compared to 51% of patients carrying the C allele in both the univariate and multivariate analysis (univariate: HR: 2.0, 95% CI: 1.04–3.99; *p* = 0.034 and multivariate: HR: 2.0, 95% CI: 1.01–3.9, *p* = 0.046).

Haplotype analyses including both *ALDH1A1* variants (rs3764435|rs168351) showed a significant association between the CA haplotype and OS (univariate: *p* = 0.034 and multivariate: *p* = 0.021). They also showed significant associations between CA and AA haplotypes and RFS in univariate (*p* = 0.004 and *p* = 0.04, respectively) and multivariate analysis (*p* = 0.001 and *p* = 0.02, respectively) (Supplementary Table S2).

### 3.3. Genetic Variants and Response

None of the evaluated SNPs were correlated with pathological response to treatment in the multivariate analysis (Table 3).

## 4. Discussion

To our knowledge, this is the first study describing the clinical significance of polymorphisms involved in the anthracycline and ifosfamide metabolic pathways in localised high-risk STS. Both drugs have high toxicity rates, especially when used in combination, and there are huge interindividual differences in grades of toxicity between patients. We found that SNPs rs3740066 and rs2273697 in *ABCC2* were significantly associated with febrile neutropenia and survival in anthracycline-treated patients. Additionally, the SNPs rs3764435 and rs168351 in *ALDH1A1* were significantly associated with survival in patients who received ifosfamide.

Doxorubicin continues to be the cornerstone in the treatment of sarcomas; however, it involves not negligible dose-limiting side effects (e.g., cardiotoxicity, myelosuppression, secondary leukaemia), and there are no validated predictive factors to identify patients at risk of these dose-limiting toxicities [38]. ABCC2 (MRP2) is an efflux transporter involved in doxorubicin exposure, which plays a central role in detoxification by secreting metabolites into bile and mediates cellular resistance to chemotherapies such as vincristine, doxorubicin and cisplatin [39]. Different polymorphisms in its encoding gene have been identified as being involved in haematological and gastrointestinal toxicities [13]. Tecza et al. conducted a study to evaluate genetic and clinical risk factors in a group of 324 breast cancer patients that received FAC chemotherapy (fluorouracil, doxorubicin and cyclophosphamide) [13]. They described a higher risk of grade 1–3 nausea for patients with CT/TT genotypes and severe neutropenia in patients with TT genotype for rs3740066. They also found the AA genotype for rs2273697 to be protective for grade 1–2 anaemia compared to AG or GG genotypes. Additionally, the GG genotype for rs2273697 has been found to be associated with decreased progression-free survival (PFS) in people with gastrointestinal stromal tumours when treated with imatinib [29], and also with worse OS and PFS in patients with mesothelioma treated with cisplatin and pemetrexed [30].

Unfortunately, there is no evidence in the literature that correlates *ABCC2* polymorphisms with the outcome and anthracycline efficacy in STS. In the present study, we found *ABCC2* rs3740066 to be associated with toxicity and decreased survival in patients with STS: patients homozygous for the T allele were more susceptible to haematological toxicity (febrile neutropenia), in line with the results reported by Tecza et al., and had shorter OS. For the other polymorphism studied in this gene, G allele carriers for rs2273697 showed longer RFS, contrary to other studies [29,30]. In addition, the TG (rs3740066|rs2273697) haplotype was also statistically significant for higher risk of febrile neutropenia. These findings are supported by previous studies that show *ABCC2* variants to have an effect on the transporter activity. *ABCC2* rs3740066 is in linkage disequilibrium with the rs717620 promoter variant that has been associated with reduced promoter activity and with lower ABCC2 mRNA levels [31,40], and rs2273697 is a nonsynonymous variant. Pharmacokinetic studies have shown that the T allele for rs3740066 was associated with higher areas under the curve for irinotecan metabolites, probably due to decreased activity of the ABCC2 transporter [27,28], and that the A allele for rs2273697 was associated with decreased systemic drug exposure [27,31,32]. These data might explain the higher risk of haematological toxicity and the association with survival observed in our sample. It should be noted that most of the published literature is focused on detecting a risk of anthracycline-related cardiotoxicity; this was not an objective in our study as cumulative anthracycline dose was low and no cardiotoxicity was reported.

ABCB1 is another multidrug efflux transporter that has been postulated to be involved in doxorubicin resistance, drug disposition, toxicity and response [4,6]. Three SNPs in this gene—rs1128503, rs2032582 and rs1045642—are in strong linkage disequilibrium [7] and are thought to play a role in drug response and disease susceptibility. In a pharmacogenetic study conducted by Caronia et al. in patients with osteosarcoma, TTT haplotype was associated with better survival [26], and the effect was higher for rs1128503, with T allele being protective for OS. In our study, we observed an association between TGT haplotype and OS, RFS and grade 3–4 anaemia, and also between CGT haplotype and OS. These allele combinations may modify the activity of the transporter and affect the therapeutic effectiveness of the chemotherapy, with an impact on survival and toxicity. It is worth mentioning that these two haplotypes are found at a very low frequency (3–7%) and, therefore, further studies are needed to establish their relevance.

Ifosfamide is one of the most useful drugs in the treatment of sarcomas; nevertheless, it is also associated with important side effects (e.g., urotoxicity, encephalopathy) that may limit its utilisation [11]. ALDH1A1 has been characterised as a determinant of cellular sensitivity to cyclophosphamide and other oxazaphosphorines [41]. It also contributes to alcohol metabolism and has been related to alcohol dependence [42]. Yao et al. published a pharmacogenetic study of 882 breast cancer patients treated with adjuvant chemotherapy including anthracyclines and ifosfamide. Patients with the AA genotype for rs3764435 and patients with AA (rs3764435|rs168351) haplotype had a higher risk of grade 3–4 haematological toxicity than C allele carriers [17]. In our sample, we could not confirm the associations of *ALDH1A1* variants with toxicity. Nevertheless, we did find a significant association between rs3764435 and CA haplotype with worse OS and RFS.

Published data on ifosfamide pharmacogenetics are scarce and, to the best of our knowledge, no studies have been previously conducted in STS. However, high expression of ALDH1A1 has been associated with resistance to chemotherapy in in vitro studies [43,44,45], and with worse disease-free survival and OS after neoadjuvant chemotherapy in breast cancer [46] The ALDH1A1 enzyme participates in the conversion process of ifosfamide to its active metabolite, by the detoxification of aldoifosfamide. Thus, altered ALDH1A1 function due to common genetic variants may affect the availability of the active metabolite, and therefore compromise patients’ survival. The rs3764435 *ALDH1A1* is an intronic variant that affects several putatively regulatory motifs, indicating that it may have an impact on the regulation of ALDH1A1 expression, as it has been reported for other intronic variants in the genome [47]. Further research is needed to delineate *ALDH1A1* variants as survival predictors.

In the present study, we have identified novel associations between *ABCC2* and *ALDH1A1* polymorphisms and toxicity and efficacy of STS treatment that add knowledge to this field of research. However, our study has certain limitations. The sample size is moderate, but unique considering STS is a rare cancer and neoadjuvant chemotherapy is not widely used. Additionally, there is inherent variability in STS histology, prognosis and chemosensitivity that is also represented in our series. In addition, DNA was isolated from peripheral blood and from FFPE tissue, and although a high correlation has been described [48], this might have influenced the genotyping results. Finally, available functionality data of the variants with significant associations in our study is not sufficient to draw definitive conclusions. Therefore, we consider this study the first hypothesis-generating pharmacogenetic study in a move towards personalizing neoadjuvant treatment in soft tissue sarcoma.

## 5. Conclusions

In the present study, we have established for the first time a relationship between *ABCC2* and *ALDH1A1* polymorphisms and toxicity and survival in high-risk STS patients receiving neoadjuvant chemotherapy. Pharmacogenetics in STS could help identify patients at lower risk of developing toxicity and those who would benefit most from neoadjuvant treatment.

## Figures and Tables

**Table 1:** Demographic and clinicopathological characteristics of high-risk soft tissue sarcoma patients (*n* = 95).

Patient and Tumour Characteristics (*n* = 95)	*n*	%
Age (years)		
Median	53	
Range	19–77	
<60	68	71.6
≥60	27	28.4
Sex		
Male	59	62.1
Female	36	37.9
ECOG * performance status		
0	34	35.8
1	40	42.1
2	4	4.2
Unknown	17	17.9
Histology		
Undifferentiated pleomorphic sarcoma	28	29.5
Synovial sarcoma	19	20.0
Spindle cell sarcoma, NOS **	15	15.8
Leiomyosarcoma	10	10.5
Myxofibrosarcoma	7	7.4
Myxoid liposarcoma	3	3.2
Pleomorphic liposarcoma	3	3.2
Malignant peripheral nerve sheath tumour	3	3.2
Others	7	7.4
Site		
Lower limb	73	76.8
Upper limb	16	16.8
Trunk	6	6.3
Chemotherapy		
Epirubicin-ifosfamide	61	64.2
High-dose ifosfamide	24	25.2
Others	10	10.5
Radiotherapy		
Neoadjuvant	40	42.1
Adjuvant	36	37.9
Neoadjuvant and adjuvant	12	12.6
No	7	7.4
Pathological response		
≥90%	35	36.8
<90%	44	56.8
Not evaluable	10	6.3

* ECOG Eastern Cooperative Oncology Group; ** NOS: not otherwise specified.

**Table 2:** Selected single nucleotide polymorphisms (SNPs) in anthracycline and ifosfamide drug pathways.

Drug Pathway/Gene Symbol	refSeq	MAF (Minor Allele)	SNP Label	Protein Label	References for Rationale
ANTHRACYCLINES					
*ABCB1*	rs1045642	0.48 (C)	c.3435T>C	p.Ile1145=	[7,14,21,22,23,24,25]
	rs2032582	0.41 (T); 0.02 (A)	c.2677T>G; c.2677T>A	p.Ser893Ala; p.Ser893Thr	[14,15,22,24]
	rs1128503	0.42 (T)	c.1236T>C	p.Gly412=	[14,26]
*ABCC2*	rs3740066	0.37 (T)	c.3972C>T	p.Ile1324=	[13,27,28]
	rs2273697	0.20 (A)	c.1249G>A	p.Val417Ile	[13,27,29,30,31,32]
*NQO1*	rs1800566	0.21 (T)	c.559C>T	p.Pro187Ser	[9,16,33]
*CBR3*	rs8133052	0.45 (A)	c.11G>A	p.Cys4Tyr	[10]
	rs1056892	0.35 (A)	c.730G>A	p.Val244Met	[10,23,34]
*SLC22A16*	rs6907567 *	0.22 (C)	c.312T>C	p.Asn104=	[29,31]
	rs12210538	0.24 (C)	c.1226T>C	p.Met409Thr	[15]
IFOSFAMIDE					
*ALDH1A1*	rs3764435	0.49 (G)	c.1434-680T>G		[17]
	rs168351	0.16 (C)	c.1434-1115T>C		[17]

*ABCB1*: ATP Binding Cassette Subfamily B Member 1, *ABCC2*: ATP Binding Cassette Subfamily C Member 2, *NQO1*: NAD(P)H Quinone Dehydrogenase 1, *CBR3*: Carbonyl Reductase 3, *SLC22A16*: Solute Carrier Family 22 Member 16, *ALDH1A1*: Aldehyde Dehydrogenase 1 Family Member A1, SNP: single nucleotide polymorphism, MAF: minor allele frequency (1000 Genomes Project, European population; accession date: 31 March 2021), refSeq: reference sequence. Label according to the accession numbers: NM_001348946.1 (*ABCB1*), NM_000392.4 (*ABCC2*), NM_000903.2 (*NQO1*), NM_001236.3 (*CBR3*), NM_033125.3 (*SLC22A16*), NM_000689.4 (*ALDH1A1*). * rs6907567 is in linkage disequilibrium with rs714368 (D’: 1.0, r2: 1.0 in European population; data from the 1000 Genomes Project).

**Table 3:** Univariate associations between genetic variants and toxicities and pathological response.

	*n*	G3-4 Anaemia *n* (%)	G3-4 Thrombo- Cytopenia *n* (%)	G3-4 Neutropenia *n* (%)	Febrile Neutropenia *n* (%)	G3-4 Transaminitis *n* (%)	Haemorrhagic Cystitis *n* (%)	Pathological Response > 90% *n* (%)
ANTHRACYCLINES								
*ABCC1*—rs1045642								
GG	19	5 (26.3%)	1 (5.3%)	8 (42.1%)	7 (36.8%)	2 (10.5%)		5 (29.4%)
AG	27	7 (25.9%)	7 (25.9%)	17 (63%)	11 (40.7%)	1 (3.8%)		10 (41.7%)
AA	7	2 (28.6%)	1 (14.3%)	3 (42.9%)	3 (42.9%)	0 (0%)		1 (14.3%)
*p*-value		1 *	0.22 *	0.323 *	1 *	0.721 *		0.413 *
*ABCC1*—rs2032582								
CC	25	8 (32%)	2 (8%)	13 (52%)	10 (40%)	2 (8%)		9 (42.9%)
CT/CA	23	4 (17.4%)	6 (26.1%)	12 (52.2%)	8 (34.8%)	1 (4.5%)		6 (27.3%)
TT/TA	5	1 (20%)	0 (0%)	2 (40%)	2 (40%)	0 (0%)		0 (0%)
*p*-value		0.528 *	0.173 *	1 *	0.917 *	1 *		0.192 *
*ABCC1*—rs1128503								
GG	24	7 (29.2%)	3 (12.5%)	12 (50%)	10 (41.7%)	2 (8.3%)		7 (33.3%)
AG	24	5 (20.8%)	5 (20.8%)	13 (54.2%)	8 (33.3%)	1 (4.3%)		8 (36.4%
AA	6	2 (33.3%)	1 (16.7%)	3 (50%)	3 (50%)	0 (0%)		1 (16.7%)
*p*-value		0.744	0.873 *	1 *	0.786 *	1 *		0.765 *
*ABCC2*—rs3740066								
CC	18	2 (11.21%)	2 (11.1%)	7 (38.7%)	5 (27.8%)	0 (0%)		5 (31.3%)
CT	24	7 (29.2%)	4 (16.7%)	13 (54.2%)	8 (33.3%)	2 (8.7%)		8 (36.4%)
TT	9	4 (44.4%)	2 (22.2%)	7 (77.8%)	7 (77.8%)	1 (11.1%)		3 (37.5%)
*p*-value		0.167 *	0.784 *	0.179 *	0.04 *	0.398		1.000 *
*ABCC2*—rs2273697								
GG	35	12 (34.3%)	8 (22.9%)	20 (57.1%)	17 (48.6%)	2 (5.9%)		13 (39.4%)
AG	15	2 (13.3%)	1 (6.7%)	6 (40%)	4 (26.7%)	1 (6.7%)		2 (15.4%)
AA	4	0 (0%)	0 (0%)	2 (50%)	0 (0%)	0 (0%)		1 (33.3%)
*p*-value		0.167 *	0.330 *	0.571 *	0.103 *	1 *		0.219 *
*NQO1—* rs1800566								
GG	34	10 (29.4%)	7 (20.6%)	21 (61.8%)	15 (44.1%)	2 (6.1%)		11 (35.5%)
AG	16	3 (18.8%)	1 (6.3%)	4 (25%)	3 (18.8%)	1 (6.3%)		2 (14.3%)
AA	4	1 (25%)	1 (25%)	3 (75%)	3 (75%)	0(0%)		3 (75.0%)
*p*-value		0.785 *	0.403 *	>0.028 *	0.058 *			0.059 *
*CBR3*—rs1056892								
GG	30	9 (30%)	5 (16.7%)	17 (56.7%)	13 (43.3%)	2 (6.9%)		12 (42.9%)
AG	18	4 (22.2%)	3 (16.7%)	8 (44.4%)	5 (27.8%)	1 (5.6%)		1 (6.7%)
AA	6	1 (16.7%)	1 (16.7%)	3 (50%)	3 (50%)	0 (0%)		5 (50%)
*p*-value		0.825 *	1 *	0.792 *	0.479 *	1 *		0.024 *
*SLC22A16*—rs6907567								
AA	29	9 (31%)	5 (17.2%)	16 (55.2%)	12 (41.4%)	1 (3.6%)		11 (42.3%)
AG	18	3 (16.7%)	4 (22.2%)	10 (55.6%)	7 (38.9%)	2 (11.1%)		3 (18.8%)
GG	7	2 (28.6%)	0 (0%)	2 (28.6%)	2 (28.6%)	0 (0%)		2 (28.6%)
*p*-value		0.564 *	0.460 *	0.478	0.926	0.71		0.256 *
IFOSFAMIDE								
*ALDH1A1*—rs3764435								
AA	23	5 (21.7%)	2 (8.7%)	9 (39.1%)	6 (26.1%)	3 (13.6%)	1 (4.5%)	9 (40.9%)
AC	30	8 (26.7%)	5 (16.7%)	15 (50%)	11 (36.9%)	0 (0%)	1 (3.3%)	10 (38.5%)
CC	18	3 (16.7%)	1 (5.6%)	7 (38.9%)	7 (38.9%)	0 (0%)	0 (0%)	6 (35.3%)
*p*-value		0.771 *	0.636	0.713 *	0.657 *		1 *	0.949 *
*ALDH1A1*—rs168351								
AA	56	14 (25%)	7 (12.5%)	24 (42.9%)	19 (33.9%)	2 (3.6%)	1 (1.8%)	17 (33.3%)
AG	14	1 (7.1%)	1 (7.1%)	6 (42.9%)	4 (28.6%)	0 (0%)	1 (7.7%)	8 (61.5%)
GG	2	1 (50%)	1 (50%)	2 (100%)	1 (50%)	1 (50%)	0 (0%)	0 (0%)
*p*-value		0.186 *	0.291 *	0.377 *	1 *		0.38 *	0.097 *

* F Fisher; G: grade; Statistically significant *p*-values are marked in bold.

**Table 4:** Univariate associations between genetic variants and overall survival (OS) and recurrence-free survival (RFS).

SNP	*n*	OS				RFS			
		Probability ± s.e * at 3-y	Probability ± s.e at 5-y	HR (95% CI)	*p*-Value	Probability ± s.e at 3-y	Probability ± s.e at 5-y	HR (95% CI)	*p*-Value
ANTHRACYCLINES									
*ABCB1*—rs1045642									
GG	16	0.83 ± 0.11	0.68 ± 0.17	1 (reference)	0.352	0.47 ± 0.13	0.47 ± 0.13	1 (reference)	0.712
AG	26	0.73 ± 0.10	0.61 ± 0.11	1.57 (0.42–5.85)		0.48 ± 0.11	0.48 ± 0.11	1.01 (0.42–2.45)	
AA	7	1.00 ± 0.00	0.80 ± 0.18	0.41 (1.04–3.99)		0.68 ± 0.19	0.45 ± 0.22	0.61 (0.16–2.31)	
*ABCB1*—rs2032582									
CC	23	0.73 ± 0.11	0.65 ± 0.12	1 (reference)	0.253	0.45 ± 0.11	0.45 ± 0.11	1 (reference)	0.59
CT/CA	21	0.83 ± 0.09	0.64 ± 0.14	1.06 (0.35–3.17)		0.49 ± 0.12	0.49 ± 0.12	0.9 (0.39–2.09)	
TT/TA	5	1.00 ± 0.00	1.00 ± 1.00	0		0.80 ± 0.18	0.53 ± 0.25	0.462 (0.1–2.08)	
*ABCB1*—rs1128503									
GG	22	0.70 ± 0.12	0.59 ± 0.14	1 (reference)	0.316	0.42 ± 0.11	0.42 ± 0.11	1 (reference)	0.552
AG	22	0.85 ± 0.08	0.70 ± 0.12	0.62 (0.2–1.89)		0.53 ± 0.12	0.53 ± 0.12	0.71 (0.3–1.65)	
AA	6	1.00 ± 0.00	0.80 ± 0.18	0.23 (0.03–1.93)		0.67 ± 0.19	0.44 ± 0.22	0.54 (0.15–1.95)	
*ABCC2*—rs3740066									
CC	15	0.87 ± 0.09	0.75 ± 0.13	1 (reference)	**0.049**	0.60 ± 0.13	0.49 ± 0.14	1 (reference)	0.471
CT	23	0.88 ± 0.08	0.80 ± 0.11	1.19 (0.29–5.02)		0.45 ± 0.12	0.45 ± 0.12	1.01 (0.4–2.53)	
TT	9	0.70 ± 0.18	0.25 ± 0.20	4.97 (1.01–24.4)		0.39 ± 0.17	NR	1.89 (0.59–5.96)	
CC/CT	38	0.88 ± 0.06	0.78 ± 0.08	4.4 (1.21–16.31)	**0.014**	0.52 ± 0.09	0.46 ± 0.09	1.86 (0.68 (5.13)	0.220
*ABCC2*—rs2273697									
GG	33	0.80 ± 0.08	0.56 ± 0.12	1 (reference)	0.092	0.47 ± 0.10	0.47 ± 0.10	1 (reference)	0.125
AG	14	0.91 ± 0.08	0.91 ± 0.08	0.33 (0.07–1.53)		0.56 ± 0.13	0.44 ± 0.15	0.91 (0.37–2.25)	
AA	3	0.33 ± 0.27	0.33 ± 0.27	2.5 (0.54–11.67)		0.33 ± 0.27	0.33 ± 0.27	3.24 (0.9–11.58)	
GG/GA	47	0.84 ± 0.06	0.68 ± 0.09	3.36 (0.74–15.2)	0.095	0.50 ± 0.08	0.45 ± 0.09	3.35 (0.97–11.51)	**0.042**
*NQO1*—rs1800566									
GG	31	0.81 ± 0.08	0.59 ± 0.11	1 (reference)	0.486	0.50 ± 0.10	0.42 ± 0.11	1 (reference)	0.325
AG	15	0.76 ± 0.12	0.76 ± 0.12	0.79 (0.25–2.55)		0.56 ± 0.14	0.56 ± 0.14	0.769 (0.3–1.96)	
AA	4	1.00 ± 0.00	1.00 ± 0.00	0		NR	NR	2.17 (0.61–7.69)	
*CBR3*—rs1056892									
GG	28	0.82 ± 0.08	0.67 ± 0.12	1 (reference)	0.33	0.41 ± 0.11	0.30 ± 0.12	1 (reference)	0.484
AG	16	0.72 ± 0.12	0.62 ± 0.14	1.8 (0.62–5.52)		0.55 ± 0.13	0.55 ± 0.13	0.87 (0.36–2.06)	
AA	6	1.00 ± 0.00	0.75 ± 0.22	0.5 (0.06–4.42)		0.67 ± 0.19	0.67 ± 0.19	0.41 (0.09–1.82)	
*SLC22A16*—rs6907567									
AA	28	0.76 ± 0.09	0.60 ± 0.11	1 (reference)	0.49	0.48 ± 0.10	0.42 ± 0.11	1 (reference)	0.279
AG	16	0.94 ± 0.06	0.94 ± 0.06	0.44 (0.09–2)		0.61 ± 0.13	0.61 ± 0.13	0.71 (0.37–2.26)	
GG	6	0.75 ± 0.22	0.38 ± 0.29	1.22 (0.27–5.67)		NR	NR	2.29 (0.73–7.22)	
*SLC22A16*—rs12210538									
AA	38	0.81 ± 0.07	0.61 ± 0.10	1 (reference)	0.163	0.48 ± 0.08	0.48 ± 0.08	1 (reference)	0.626
AG	8	1.00 ± 0.00	1.00 ± 0.00	0		0.67 ± 0.20	0.33 ± 0.26	0.58 (0.17–1.96)	
GG	4	0.50 ± 0.25	0.50 ± 0.25	1.65 (0.37–7.42)		0.36 ± 0.28	0.36 ± 0.28	1.23 (0.28–5.31)	
IFOSFAMIDE									
*ALDH1A1*—rs3764435									
AA	22	0.69 ± 0.11	0.38 ± 0.09	1 (reference)	0.062	0.25 ± 0.10	0.25 ± 0.10		0.085
AC	29	0.76 ± 0.09	0.65 ± 0.10	0.56 (0.23–1.33)		0.50 ± 0.10	0.50 ± 0.10		
CC	17	0.88 ± 0.08	0.80 ± 0.11	0.24 (0.06–0.88)		0.63 ± 0.12	0.53 ± 0.12		
AC/CC	46	0.81 ± 0.06	0.71 ± 0.08	2.29 (1.02–5.17)	**0.038**	0.55 ± 0.08	0.51 ± 0.08	2.04 (1.04–3.99)	**0.034**
*ALDH1A1*—rs168351									
AA	53	0.81 ± 0.06	0.63 ± 0.08	1 (reference)	**0.015**	0.46 ± 0.07	0.43 ± 0.07	1 (reference)	0.306
AG	14	0.67 ± 0.14	0.56 ± 0.15	1.64 (0.65–4.17)		0.46 ± 0.16	0.46 ± 0.16	1.14 (0.49–2.61)	
GG	1	NR	NR	11.8 (1.4–99.7)		NR	NR	4.31 (0.56–33.01)	
AG/GG	15	0.62 ± 0.14	0.52 ± 0.15	1.86 (0.77–4.5)	0.16	0.42 ± 0.15	0.42 ± 0.15	1.25 (0.57–2.75)	0.575

The statistically significant *p*-values are marked in bold; * s.e: standard error; NR: not reached.

## Data Availability

The data presented in this study are not publicly available due to ethical committee regulations. The data are available on request from the corresponding authors.

## Supplementary Materials

The following supporting information can be downloaded at: https://www.mdpi.com/article/10.3390/jpm12040618/s1, Supplementary Table S1 (Haplotype association analysis of ABCC2, ABCB1 and ALDH1A1 polymorphisms with toxicity/response); Supplementary Table S2 (Haplotype association analysis of *ABCC2*, *ABCB1* and *ALDH1A1* polymorphisms with survival).
