# Supplementary material for: Pharmacogenetic Profiling in High-Risk Soft Tissue Sarcomas Treated with Neoadjuvant Chemotherapy
Source: J Pers Med. 2022 Apr 11;12(4):618. doi: 10.3390/jpm12040618 (PMC9024670; doi:10.3390/jpm12040618)
Supplement: Supplementary file 1 [file jpm-12-00618-s001.zip › Supplementary Table S1_AVirgili.pdf]

Supplementary Table S1. Haplotype association analysis of *ABCC2*, *ABCB1* and *ALDH1A1* polymorphisms with toxicity/response.

		**Anthracycline pathway**									**Ifosfamide pathway**			
		ABCB1 (rs1128503|rs2032582|rs1045642)					*ABCC2* (rs3740066|rs2273697)				*ALDH1A1* (rs3764435|rs168351)			
		TTT	TGT	CGT	TGC	CGC	TA	CA	TG	CG	CG	AG	CA	AA
**Grade 3-4 anaemia**	Frequency affected	0.244	0.075	0.074	0.003	0.604	0.002	0.075	0.575	0.348	0.003	0.091	0.435	0.472
	Frequency unaffected	0.303	0.013	0.066	0.013	0.605	0.016	0.260	0.339	0.384	0.013	0.105	0.459	0.422
	***p*-value** (univariate)	0.555	0.098	0.888	0.643	0.999	0.580	**0.046**	**0.034**	0.742	0.619	0.818	0.804	0.621
	***p*-value** (multivariate)	0.258	**0.02**	0.917	0.601	0.549	0.79	0.165	0.104	0.683	0.481	0.846	0.618	0.399
**Grade 3-4 thrombo-cytopenia**	Frequency affected	0.379	0.01	0.111	0	0.5	0	0.063	0.5	0.438	0.006	0.057	0.432	0.506
	Frequency unaffected	0.269	0.033	0.060	0.013	0.626	0.015	0.241	0.381	0.364	0.012	0.107	0.457	0.424
	***p*-value** (univariate)	0.344	0.584	0.432	0.633	0.320	0.627	0.109	0.371	0.574	0.834	0.527	0.850	0.536
	***p*-value** (multivariate)	0.989	0.786	0.361	0.995	0.914	0.996	0.237	0.219	0.938	0.708	0.508	0.609	0.318
**Grade 3-4 neutropenia**	Frequency affected	0.288	0.038	0.092	0.001	0.580	0.021	0.164	0.479	0.336	0.021	0.108	0.447	0.424
	Frequency unaffected	0.287	0.020	0.043	0.020	0.630	0.002	0.269	0.310	0.419	0.004	0.096	0.459	0.441
	***p*-value** (univariate)	0.989	0.587	0.314	0.345	0.601	0.380	0.197	0.083	0.390	0.331	0.816	0.888	0.838
	***p*-value** (multivariate)	0.981	0.411	0.247	0.511	0.422	0.695	0.398	0.104	0.227	0.501	0.787	0.869	0.877
**Febrile neutropenia**	Frequency affected	0.289	0.028	0.099	0.0009	0.584	0.026	0.074	0.524	0.376	0.025	0.100	0.496	0.379
	Frequency unaffected	0.287	0.031	0.050	0.016	0.617	0.003	0.303	0.319	0.374	0.004	0.102	0.432	0.461
	***p*-value** (univariate)	0.982	0.931	0.331	0.447	0.738	0.305	**0.006**	**0.040**	0.984	0.262	0.968	0.469	0.348
	***p*-value** (multivariate)	0.832	0.781	0.240	0.621	0.652	0.474	**0.035**	**0.040**	0.697	0.487	0.93	0.574	0.429
**Vomiting**	Frequency affected	0	0	0	0	1	0.012	0.208	0.398	0.382	0	0	0	1
	Frequency unaffected	0.293	0.030	0.070	0.011	0.597	0.030	0.829	0.048	1.062	0.011	0.103	0.460	0.425
	***p*-value** (univariate)	0.365	0.804	0.699	0.883	0.248	0.862	0.363	0.827	0.303	0.881	0.632	0.194	0.104
	***p*-value** (multivariate)	0.996	0.999	0.988	0.995	0.995	0.976	0.491	0.833	0.57	1	0.997	0.995	0.993
**Grade 3-4 transamititis**	Frequency affected	0.167	0	0	0	0.833	0.009	0.158	0.658	0.175	0	0.333	0	0.667
	Frequency unaffected	0.290	0.032	0.074	0.011	0.593	0.013	0.221	0.381	0.385	0.012	0.085	0.481	0.422
	***p*-value** (univariate)	0.514	0.657	0.489	0.794	0.241	0.928	0.716	0.178	0.303	0.790	**0.044**	**0.021**	0.237
	***p*-value** (multivariate)	0.896	0.995	0.987	0.995	0.376	0.924	0.609	0.283	0.445	0.997	0.121	0.994	0.424
**Haemorrhagic cystitis**	Frequency affected										0	0.25	0.25	0.5
	Frequency unaffected										0.011	0.091	0.467	0.431
	***p*-value** (univariate)										0.829	0.289	0.392	0.783
	***p*-value** (multivariate)										0.997	0.387	0.463	0.804
**Pathological response >90%**	Frequency affected	0.242	0.068	0.065	0.002	0.623	0.031	0.094	0.406	0.469	0.024	0.136	0.416	0.424
	Frequency unaffected	0.339	0	0.065	0.015	0.582	0.003	0.247	0.397	0.353	0.004	0.084	0.472	0.441
	***P*-value** (univariate)	0.331	**0.033**	0.996	0.567	0.698	0.264	**0.077**	0.928	0.281	0.288	0.342	0.538	0.847
	***P*-value** (multivariate)	0.269	0.995	0.810	0.528	0.503	0.511	0.2	0.783	0.471	0.544	0.217	0.415	0.936

The statistically significant *p*-values are marked in bold.
