# Supplementary material for: Pharmacogenetic Profiling in High-Risk Soft Tissue Sarcomas Treated with Neoadjuvant Chemotherapy
Source: J Pers Med. 2022 Apr 11;12(4):618. doi: 10.3390/jpm12040618 (PMC9024670; doi:10.3390/jpm12040618)
Supplement: Supplementary file 1 [file jpm-12-00618-s001.zip › Supplementary Table S2_AVirgili.pdf]

Supplementary Table S2. Haplotype association analysis of *ABCC2*, *ABCB1* and *ALDH1A1* polymorphisms with survival.

	**Overall survival**		**Recurrence-free survival**	
**Haplotype**	***p*-value univariate**	***p*-value multivariate**	***p*-value univariate**	***p*-value multivariate**
**Anthracycline pathway**				
***ABCB1* (rs1128503|rs2032582|rs10456429)**				
**TTT**	0.243	0.083	0.394	0.135
**TTC**	0.459	0.498	0.540	0.545
**TGT**	0.059	**<0.001**	**<0.001**	**0.001**
**CGT**	**0.042**	**0.047**	0.109	0.112
**CGC**	0.171	0.053	0.263	0.092
***ABCC2* (rs3740066|rs2273697)**				
**TA**	0.815	0.988	0.402	0.275
**CA**	0.544	0.835	0.486	0.869
**TG**	0.091	0.185	0.095	0.193
**CG**	0.216	0.224	0.167	0.133
**Ifosfamide pathway**				
***ALDH1A1* (rs3764435|rs168351)**				
**CG**	0.313	0.579	0.363	0.665
**AG**	0.405	0.369	0.284	0.228
**CA**	**0.034**	**0.021**	**0.004**	**0.001**
**AA**	0.139	0.094	**0.040**	**0.020**

The statistically significant *p*-values are marked in bold.
